# Frequency of viral infections in adolescent and adult in-patient Ethiopians with acute leukemia at presentation to a tertiary care teaching hospital: a cross-sectional study

**DOI:** 10.1186/s13027-023-00519-6

**Published:** 2023-07-12

**Authors:** Jemal Alemu, Balako Gumi, Aster Tsegaye, Abdulaziz Abubeker, Fisihatsion Tadesse, Abel Shewaye, Ziyada Rahimeto, Adane Mihret, Andargachew Mulu, Amha Gebremedhin, Rawleigh Howe

**Affiliations:** 1 Department of Medical Laboratory Sciences, College of Health Sciences, Addis Ababa University, Addis, Ababa Ethiopia; 2 Armauer Hansen Research Institute, Addis Ababa, Ethiopia; 3 Aklilu Lemma Institute of Pathobiology, Addis Ababa University, Addis Ababa, Ethiopia; 4 Department of Internal Medicine, College of Health Sciences, Addis Ababa University, Addis, Ababa Ethiopia; 5 Department of Laboratory, ALERT Hospital, Addis Ababa, Ethiopia; 6 Department of Microbiology, Immunology and Parasitology, College of Health Sciences, Addis Ababa University, Addis Ababa, Ethiopia

**Keywords:** Viral infections, HBV, HCV, HIV, OBI, Acute leukemia, Ethiopia

## Abstract

**Background:**

Leukemic patients are prone to infectious agents such as viruses due to dysregulated immune system resulting from infiltration of the bone marrow by malignant cells, chronic stimulation, reactivation of some viruses and viral pathogenicity as well as rarely from acquisition of a new infections leading to severe complications. However, the prevalence of these infections has not been systematically documented in resource-limited settings such as Ethiopia.

**Objective:**

To determine the prevalence of HBV, HCV, and HIV among adult and adolescent in-patients with acute leukemia before the administration of chemotherapy, at the Tikur Anbessa Specialized Hospital (TASH) in Addis Ababa, Ethiopia.

**Methods:**

A cross sectional study was conducted on 176 adult and adolescent inpatient Ethiopians, who were diagnosed with acute leukemia from April 2019 to June 2021. Socio-demographic characteristics and relevant clinical data were collected. Peripheral blood samples were collected and tested for HBV, HIV, and HCV using Enzyme-Linked Immunosorbent Assay (ELISA) and real-time PCR. Chi-square tests were used to assess associations between variables.

**Results:**

Of the 176 patients, 109(62%) were males. The median age was 25[IQR,18–35] yr, with a range from 13 to 76 year. The prevalence of HBV (positivity for HBsAg plus HBV DNA), HCV and HIV was 21.6%, 1.7%, and 1.7%, respectively. HBsAg was positive in 19 cases (10.8%). Among 157 HBsAg negative patients, 52(33.1%) were positive for Anti-HBcAg; of these seropositive cases, 47.5% were positive for HBV DNA. Most DNA positive, HBsAg negative cases (79.0%) had DNA concentrations below 200 IU/ml indicating true occult HBV infection (OBI). Of the 176 cases, 122 had a history of blood transfusions, but no statistically significant association was found between HBV infection and blood product transfusion history (P = 0.963).

**Conclusions:**

The prevalence of HBV, HIV and HCV in patients with acute leukemia was similar to the national prevalence level of these infections. Given the HBsAg positivity and the high prevalence of occult hepatitis B infection in our study, these patients may be at increased risk for chemotherapy related hepatitis flares. Hence, clinicians caring these patients are strongly advised to screen their patients for HBV and also for HIV and HCV infections routinely.

## Background

Acute leukemia (AL) is caused by genetic lesions within hematological progenitor cells [1]. Viral infections are common in hematological malignancies including leukemia due to immunocompromise mainly related to bone marrow infiltration and crowding, but also from the effects of immunosuppressive chemotherapy [2–6].

Blood-borne viruses such as Hepatitis B virus (HBV), Hepatitis C virus (HCV) and Human immuno-deficiency virus (HIV), are common in developing countries and may affect patients with hematological malignancies and potentially complicate their treatment outcomes [7, 8]. Hepatitis B and C infection in hematological patients are reportedly prone to reactivation of latent infections and may also from new infection [7]. Several studies have reported associations between HBV/ HCV with hematological malignancies [9–11].

Moreover, higher prevalences of co-infections of these viruses have been noted among cancer patients [12]. However, little is known about the frequency of blood born virus infections in acute leukemia patients in resource limited settings like Ethiopia. This study, therefore, aimed to determine the prevalence of blood born viruses namely HBV, HIV and HCV before initiation of chemotherapy in acute leukemia adolescent and adult patients admitted in a tertiary care teaching hospital in Addis Ababa, Ethiopia.

## Materials and methods

### Hospital setting and patients

A cross-sectional study was conducted on those adolescent and adult patients (aged 13 years and above) diagnosed with acute leukemias who were admitted at the hematology clinic of Tikur Anbessa Specialized Hospital (TASH) from April 2019 to June 2021. TASH was established in 1972, has more than 700 beds and more than 700 health professionals. It is the main referral hospital in the country to diagnose, treat and manage cancer patients including hematological malignancies. Historically the adult Hematology clinic serves patients aged 13 years and above as well as provides service for more than 20,000 patients annually [13].

A standardized form was used to collect socio-demographic characteristics and relevant clinical data. Patients were recruited at the time of admission before starting any type of chemotherapy. Following informed consent and/or assent for those aged 13 to 17 years, peripheral blood samples were collected from study participants. Three ml of blood was drawn in EDTA tube and centrifuged for plasma separation; the plasma was stored at -70 ^0^ C until analysis.

### HBV, HIV and HCV Testing

The stored plasma samples were thawed and used for ELISA, rapid and PCR tests. HBsAg was detected by enzyme immunoassay (HBsAg ELISA-Beijing WantaiBiological Pharmacy Enterprise Co. Ltd, China), HBsAg negative samples were tested for both IgM and IgG by anti-HBcAg ELISA kits (Monolisa Anti-HBc PLUS, Bio-Rad, France) at the Armauer Hansen Research Institute (AHRI). Anti-HBcAg positive samples were tested for HBV DNA by PCR. DNA was extracted, amplified, and detected from 200 µl plasma samples using a commercially available Real-time PCR platform (Abbott m2000rt) with a lower detection limit < 1.18 Log IU/ml genome equivalent to 15 IU/ml at ALERT hospital laboratory to determine Occult hepatitis B infection (OBI).

HIV was screened by Enzyme linked immunosorbent assay (Micro ELISA–HIV Ag & Ab, J. Mitra & Co. Pvt. Ltd., New Delhi, India). Positive samples were repeated with rapid tests using the Ethiopian national algorithm: CHEMBIO, Chembio Diagnostic Systems. Inc, (Medford, NY, USA); ABON, Abon Biopharm Co. Ltd, P.R. (Hangzhou, China); SD HIV 1/2 3.0, Standard Diagnostics, Inc (Republic of Korea).

HCV was screened by Enzyme linked immune assay (anti-HCV ELISA–Beijing Wantai Biological Pharmacy Enterprise Co. Ltd, China) and positive samples checked with Wondfo one step rapid Ab assay for HCV, Guangzhou Wondfo Biotech Co., Ltd, China. All the tests were performed according to the manufacturer’s instruction. All quality control steps were handled as per the standard operating procedures.

### Statistical analysis

Data was entered into an excel spread sheet, transported into SPSS version 25 statistical software and analyzed by descriptive statistics including mean, median, range, standard deviation and percentage. The Chi square test was used for studying the association between various variables. A P-value < 0.05 was considered statistically significant.

## Results

A total of 176 adolescent and adult acute leukemia patients were included in this study and 109/176 (61.9%) were males. The median age of the study participants was 25[IQR 18–35] years; age range (13–76) years. Most, 59.7%, were in the age group of 18–39 years (105/176), while 19.9% of the study participants were grouped within the 13–17 age category, and 5.1% were of the 60–89 age group. The majority of the cases (83/160, 51.9%) were acute lymphoblastic leukemia (ALL) cases based on Leukemia diagnosis (Table 1). Two patients were previously diagnosed with chronic myelogenous leukemia undergoing acute blast crisis [14, 15].

**Table 1:** Baseline demographic and clinical characteristics of adolescent and adult acute leukemia patients, n = 176

Variables	Count (%)
Age	
13–17 18–39 40–59 60 and above	35(19.9) 105(59.7) 27(15.3) 9(5.1)
Sex	
Male Female	109(61.9) 67(38.1)
Clinical Assessment	
Complex of anemia Pancytopenia Leukocytosis Blast crisis Other	112(71.3) 10(6.4) 5(3.2) 2(1.3) 28(17.8)
Leukemia Diagnosis	
ALL AML Blast crisis* Mixed Leukemia Not specified	83(51.9) 67(41.9) 2(1.2) 1(0.6) 7(4.4)

# Abbreviations used: ALL (Acute Lymphoblastic Leukemia), AML (Acute Myeloblastic Leukemia)

* Chronic myelogenous leukemia undergoing accumulation of undifferentiated blasts

Among 176 cases, detectable HBV (either HBsAg positive or Occult HBV infection (OBI)) was detected in 21.6% (38/176) of the participants. Males were more infected than females, 22.9% (25/109) versus 19.4% (13/67), respectively; however, these differences were not statistically significant (P = 0.580). Patients in the age group of 18–39 years were the most commonly infected comprising 24.8% (26/105), and those in the age group of 60 and above, 11% (1/9), were the least affected. These differences were not statistically significant (p = 0.590).

HBsAg was detected in 19/176 (10.8%) of the cases. Among the 157 HBsAg negative samples, we further observed that 52/157 (33.1%) were positive for anti-HBcAg. Of these 52, DNA extraction, amplification and detection was conducted in 40 and viral DNA was detected in 47.5% (19/40) of the analyzed samples. This represented 10.8% of all samples. From these 19 samples, 15 had HBV DNA concentration below 200 IU/ml, indicating true OBI. Among DNA positive cases, 16/19(84,2%) were males and this gender discrepancy was statistically significant (p = 0.034).

Most of the cases with positive DNA were in the 18–39 year old age group, 73.7% (14/19). In contrast, 15.8% (3/19), 5.2% (1/19) and 5.2% (1/19) of the study subjects were among the 13–17, 40–59, as well as 60 and above age groups, respectively (p = 0.728).

From the total cases, 145 had transfusion histories recorded. Of these, 84.1% (122/145) had been transfused with blood or blood products. Among all cases with complete records, 21.4% (31/145) were positive for HBV (either HBsAg positive or DNA positive). Of the 31 positive cases 26 had been transfused. Of those who were HBV positive and had been transfused, 12 (46.2%) had occult infection, whereas 14 (53.8%) were HBsAg positive. There was no statistically significant association between HBV infection and previous transfusion history (P = 0.963) (Table 2).

**Table 2:** Cases with available previous transfusion history data before Chemotherapy, n = 145

Laboratory results	Transfusion status			P value*
	Transfused cases (122)	Non transfused cases (23)	Total	
	Count (%)	Count (%)	Count (%)	
HBsAg pos	14(11.5)	1(4.3)	15(10.3)	0.303
HBV-DNA pos	12(9.8)	4(17.4)	16(11)	0.289
Total HBV (HBsAg pos and HBV-DNA pos)	26(21.3)	5(21.7)	31(21.4)	.0963

# Abbreviation used: HBV (Hepatitis B virus), HBsAg (Hepatitis B surface antigen), pos (positive)

* Chi-square was used to analyze the association

HIV seroprevalence was 1.7% (3/176). All the three infected cases were female (p = 0.026); two were in the age category of 40–59, and one in the 60 and above age group. HCV sero-prevalence was 1.7% (3/176) and all the three infected cases were male (p = 0.171), two were in the 18–39 age category, and one in 40–59 group (Table 3).

**Table 3:** HBV, HCV and HIV distribution in gender of acute leukemia cases

Laboratory results	Gender			
	Male	Female	Total	P value*
	Count (%)	Count (%)	Count (%)	
Total HBV (HBsAg pos and HBV-DNA pos)	25(22.9)	13(19)	38(21.6)	0.580
HBsAg pos	9(8.3)	10(14.9)	19(10.8)	0.166
HBsAg neg, Anti-HBcAg pos and HBV-DNA pos	16(14.8)	3(4.5)	19(10.8)	0.034
HBsAg neg, Anti-HBcAg pos and HBV-DNA neg	10(9.2)	11(16.4)	21(11.9)	0.150
Ag and Anti-HIV pos	0(0)	3(4.5)	3(1.7)	0.026
Anti-HCV pos	3(2.8)	0(0)	3(1.7)	0.171

# Abbreviation used: HBV (Hepatitis B virus), HIV (Human Immunodeficiency Virus), HCV (Hepatitis C virus), HBsAg (Hepatitis B surface antigen), HBcAg (Hepatitis B core antigen), pos(positive), neg(negative), Ag (Antigen),

* Chi-square was used to analyze the association

The prevalence of viral infections is consistent with pooled results reported by systematic reviews of HBV and HCV prevalences at the population level in Ethiopia [16] as well as HIV in Addis Ababa [17]. To probe the possible relationship of viral infection and leukemia further, we determined whether there were associations between viral infection and subtypes of leukemia. As shown in Table 4, none of the viruses were associated with subtypes of acute leukemia.

**Table 4:** Association of HBV, HCV and HIV with acute leukemias, n = 160

	Leukemia Diagnosis						Total Count (%)	P value*
		ALL Count (%)	AML Count (%)	Blast Crisis Count (%)	Mixed Leukemia Count (%)	Non-Specified Count (%)		
HBsAg	pos	7(8.4)	11(16.4)	0(0)	0(0)	0(0)	18(11.3)	0.156
	Neg	76(91.6)	56(83.6)	2(100)	1(100)	7(100)	142(88.8)	
HBV-DNA	Pos	8(9.6)	7(8.4)	0(0)	0(0)	2(28.6)	17(10.6)	0.603
	Neg	75(90.4)	60(89.6)	2(100)	1(100)	5(71.4)	143(89.4	
Total HBV	Pos	15(18.1)	18(26.9)	0(0)	0(0)	2(28.6)	35(21.9)	0.428
	Neg	68(81.9)	49(73.1)	2(100)	1(100)	5(71.4)	125(78.1)	
HCV	Pos	2(2.4)	1(1.5)	0(0)	0(0)	0(0)	3(1.9)	0.830
	Neg	81(97.6)	66(98.5)	2(100)	1(100)	7(100)	157(98.1)	
HIV	Pos	3(3.6)	0(0)	0(0)	0(0)	0(0)	3(1.9)	0.242
	Neg	80(96.4)	67(100)	2(100)	1(100)	7(100)	157(98.1)	

# Abbreviations used: HBV (Hepatitis B virus), HBsAg (Hepatitis B surface antigen), HCV (Hepatitis C virus), Pos (positive), HIV (Human Immunodeficiency Virus), Neg (negative), ALL (Acute Lymphoblastic Leukemia), AML (Acute Myeloblastic Leukemia)

* p values according to Chi-square test

## Discussion

In this study, we analyzed the prevalence of three chronic viral infections, HBV, HIV and HCV in patients with acute leukemia admitted to a tertiary care teaching hospital in Ethiopia. Our study indicates that HBV (included occult infection) was the most prevalent viral infection in these patients. HBV infection is a major public health problem worldwide particularly in low- and middle- income countries [18–24].

In Ethiopia the prevalence of HBsAg has reportedly ranged from 3.4 to 11.9% [25–33]. Several factors have been identified to play role for the increased burden of HBV in resource constrained settings. These include under diagnosis, inadequate system for prevention (such as vaccination), and suboptimal contact tracing and treatment and care of affected individuals. Additional factors include poor awareness of the community about infections and their transmission, treatment inaccessibility and unaffordability and in some occasions limited awareness of the health professionals of the current treatment guidelines. Increased internal and external migration rates, administrative and regulatory issues that hinder commitment of resource mobilization at the national health regulatory level, and weak coordination with international partners, also play role [34–39]. In our study, HBV was detected in 38(21.6%) of our cases, out of which 19(10.8%) were positive for HBsAg and the remaining 19(10.8%) were positive for HBV DNA test. The later were negative for HBsAg.

These findings are consistent with studies of leukemia patients reported from Korea, Iraq and Iran [40–42] as well as consistent with prevalence studies in Ethiopia of HBsAg [28, 30, 33]. HBV DNA has been detected in peripheral blood, even when levels of HBsAg or anti-HBcAg are undetectable. When HBsAg is negative, detectable HBV DNA is termed Occult HBV infection (OBI).

Because blood levels may be low (< 200 IU) (true OBI) and the liver is also relatively inaccessible [43–45]. Occult HBV infection can be challenging to detect, particularly in immunocompromised patients such as in patients with leukemia [46–48]. OBI prevalence is variable depending on the nature of the study [43, 45, 49–51] hence the HBV DNA test is recommended as a preferred reliable marker for identifying HBV infection [45]. In leukemia patients, of the growing concerns of chemotherapy-related flares of HBV, which can be fatal, have led to recommendations for DNA testing [52–55]. Indeed, in our study, 19 (10.8%) of the cases were positive for occult infection, similar to studies conducted in China and India [23, 48].

It is notable that we observed male predominance of occult HBV infection in our study population (P = 0.034). However, the difference in non-occult HBsAg positivity was not statistically significant between males and females (P = 0.166). Contrary to our findings, multiple previous studies have reported higher prevalence of HBsAg rates among males [40, 41, 56–58]. The pathogenesis of HBV infection due to gender difference may be related to sex exposure risks such as occupational, travel, social and life style differences [41], as well as due to immune effects related to reproductive hormones. Moreover, stimulatory effects of androgen in males on interleukin-6 (IL-6) enhances HBV gene expression /transcription and decreases viral immune response such as apoptosis which may also be associated with oncogenesis, whereas estrogen influenced IL-6 production in females resulting in mediating apoptosis. In addition, estrogen inhibits HBV RNA transcription [59–65]. The finding in our study that occult but not non-occult HBV infection was more prominent among males in this leukemia cohort would be consistent with an immune based mechanism related to reproductive hormones.

A possible explanation underlying occult HBV infection in this leukemia cohort may be related to the use of transfusions of blood and/or blood products, known to transmit HBV infection [40, 42]. Although the majority of patients in our study had been transfused with blood products, we did not find a statistically significant association between either total or occult HBV infection and previous transfusion history (P > 0.05), a finding in line with other reports of HBV positivity [23]. Further studies may reveal whether patients such as these are at risk for hepatitis flares in the context of chemotherapy treatment.

Regarding HIV and HCV seroprevalence in our patients, HIV seroprevalence was 1.7% (3/176), and all were females, greater than 40 years of age. Similar to our study, HIV positivity has been reported in patients with hematological malignancies elsewhere [66, 67]. Although HIV infection in leukemia patients has not been systematically reported in Ethiopia, many studies in other population groups have indicated that females are affected more than males in Ethiopia [68–70], with some exceptions noted [8]. The gender difference in HIV infection may be related to the greater mucosal surface area exposure in females during sexual intercourse as well as under diagnosis and screening related to asymptomatic presentation of other sexually transmitted diseases in women [71, 72].

Our study showed that HCV sero-prevalence was 1.7% (3/176) and all the three cases were males, two were in the 18–39-year age category, and one in 40–59. These findings are consistent with overall reported seroprevalence of HCV in Ethiopia in multiple studies, ranging from 0.6 to 6% [26, 27, 73, 74].

We did not observe differences in HBV, HCV or HIV prevalence among AML and ALL subtypes of leukemia. This contrasts with the findings of study who reported that AML patients had higher prevalences of HBV viruses in Korea [40]. This could relate to age differences in the presentation of both leukemia and HBV infection [9, 40].

### Limitations

Due to resource limitation, some of anti-HBcAg positive samples were not evaluated for DNA, however, sufficient information were obtained from the analyzed samples. Moreover, because we did not include a control population of non-leukemic patients, we cannot be certain that these viral infection prevalences are identical to those of the general population; however, they are consistent with previous systematic reviews of multiple population-based studies in Ethiopia.

## Conclusions

This is the first study in Ethiopia examining the prevalence of viral infections among leukemia patients. Our study showed that the prevalence of HBV, HIV and HCV among leukemia patients is similar to the national prevalence reported in the general population. Of particular concern is the high prevalence of HBsAg positive and occult hepatitis B, which collectively comprised a significant proportion of leukemia patients. Given that these patients are at risk for chemotherapy related complications, clinicians are strongly advised to screen their patients for HBV, HIV and HCV infections.

## Data Availability

The data that support the findings of this study are available on request from the corresponding author. The data are not publicly available due to privacy or ethical restrictions.

## Abbreviations

AHRI: Armauer Hansen Research Institute
AL: Acute Leukemia
ALERT: All African Leprosy Rehabilit- ation Training Center
ALL: Acute Lymphoblastic Leukemia
AML: Acute Myeloblastic Leukemia
DNA: Deoxyribonuceic acid
EDTA: Ethylenediaminetetraacetic acid
ELISA: Enzyme-linkedimmunosorbent assay
HBsAg: Hepatitis B surface antigen
HBV: Hepatitis B Virus
HCV: Hepatitis C virus
HIV: Human Immunodeficiency Virus
IL: Interleukin
IQR: Interquartile Range
OBI: Occult hepatitis B Infection
PCR: Polymerase Chain Reaction
SPSS: Statistical Package for the Social Sciences
TASH: Tikur Anbessa Specialized Hospital

## References

[1] Fiegl M. Epidemiology, pathogenesis, and etiology of acute leukemia. Handbook of acute leukemia. Springer; 2016. pp. 3–13.

[2] Chandran R, Hakki M, Spurgeon S. Infections in leukemia. Sepsis-An Ongoing and Significant Challenge. 2012:334 – 68.

[3] Andersen MH (2014). The targeting of immunosuppressive mechanisms in hematological malignancies. Leukemia.

[4] Balducci L, Dolan D (2015). Chronic lymphocytic leukemia in the elderly: epidemiology and proposed patient-related approach. Cancer Control.

[5] Teh BW, Tam CS, Handunnetti S, Worth LJ, Slavin MA (2018). Infections in patients with chronic lymphocytic leukaemia: mitigating risk in the era of targeted therapies. Blood Rev.

[6] Safdar A, Armstrong D (2011). Infections in patients with hematologic neoplasms and hematopoietic stem cell transplantation: neutropenia, humoral, and splenic defects. Clin Infect Dis.

[7] Busca A (2012). Viral infections in patients with hematological malignancies. Leuk supplements.

[8] Biadgo B, Shiferaw E, Woldu B, Alene KA, Melku M (2017). Transfusion-transmissible viral infections among blood donors at the north Gondar district blood bank, northwest Ethiopia: a three year retrospective study. PLoS ONE.

[9] Anderson LA, Pfeiffer R, Warren JL, Landgren O, Gadalla S, Berndt SI (2008). Hematopoietic malignancies associated with viral and alcoholic hepatitis. Cancer Epidemiol Biomarkers Prev.

[10] Matsuo K, Kusano A, Sugumar A, Nakamura S, Tajima K, Mueller NE (2004). Effect of hepatitis C virus infection on the risk of non-hodgkin’s lymphoma: a meta‐analysis of epidemiological studies. Cancer Sci.

[11] Park SC, Jeong SH, Kim J, Han CJ, Kim YC, Choi KS (2008). High prevalence of hepatitis B virus infection in patients with B-cell non‐Hodgkin’s lymphoma in Korea. J Med Virol.

[12] Kocabas E, Aksaray N, Alhan E, Tanyeli A, Koksal F, Yarkin F (1997). Hepatitis B and C virus infections in turkish children with cancer. Eur J Epidemiol.

[13] Tikur Anbessa Specialized Hospital (TASH). 2023. http://www.aau.edu.et/chs/tikur-anbessa-specialized-hospital/background-of-tikur-anbessa-hospital.Accessed 8 Apr 2023.

[14] Chereda B, Melo JV (2015). Natural course and biology of CML. Ann Hematol.

[15] Perrotti D, Jamieson C, Goldman J, Skorski T (2010). Chronic myeloid leukemia: mechanisms of blastic transformation. J Clin Investig.

[16] Belyhun Y, Maier M, Mulu A, Diro E, Liebert UG (2016). Hepatitis viruses in Ethiopia: a systematic review and meta-analysis. BMC Infect Dis.

[17] Adal M (2019). Systematic review on HIV situation in Addis Ababa, Ethiopia. BMC Public Health.

[18] Liaw YF, Brunetto MR, Hadziyannis S (2010). The natural history of chronic HBV infection and geographical differences. Antivir Ther.

[19] Lavanchy D (2004). Hepatitis B virus epidemiology, disease burden, treatment, and current and emerging prevention and control measures. J Viral Hepatitis.

[20] Ott J, Stevens G, Groeger J, Wiersma S (2012). Global epidemiology of hepatitis B virus infection: new estimates of age-specific HBsAg seroprevalence and endemicity. Vaccine.

[21] Lalazar G, Rund D, Shouval D (2007). Screening, prevention and treatment of viral hepatitis B reactivation in patients with haematological malignancies. Br J Haematol.

[22] Barclay S, Pol S, Mutimer D, Benhamou Y, Mills PR, Hayes PC (2008). The management of chronic hepatitis B in the immunocompromised patient: recommendations from a single topic meeting. J Clin Virol.

[23] Sodhi JS, Raja W, Zargar SA, Javid G, Aejaz S, Ahmad M (2015). Screening for occult and overt hepatitis B virus infection in patients of cancer before receiving chemotherapy: looking beyond HBsAg testing. Int J Adv Res.

[24] Dan YY, Aung MO, Lim SG (2008). The economics of treating chronic hepatitis B in Asia. Hep Intl.

[25] Abebe A, Nokes D, Dejene A, Enquselassie F, Messele T, Cutts F (2003). Seroepidemiology of hepatitis B virus in Addis Ababa, Ethiopia: transmission patterns and vaccine control. Epidemiol Infect.

[26] Woldegiorgis AE, Erku W, Medhin G, Berhe N, Legesse M (2019). Community-based sero-prevalence of hepatitis B and C infections in South Omo Zone, Southern Ethiopia. PLoS ONE.

[27] Kumalo A, Teklu T, Demisse T, Anjulo A (2022). Undiagnosed seroprevalence of Hepatitis B and C Virus Infections in the community of Wolaita Zone, Southern Ethiopia. Hepatic Medicine: Evidence and Research.

[28] Belay AS, Abateneh DD, Yehualashet SS, Kebede KM (2020). Hepatitis B virus infection and associated factors among adults in Southwest Ethiopia: community-based cross-sectional study. Int J Gen Med.

[29] Tadesse M, Tafesse G, Hajare ST, Chauhan NM (2022). Assessment of prevalence of Hepatitis B virus and its associated factors among pregnant women from Wolaita Sodo, Ethiopia. J Clin Virol Plus.

[30] Wondmagegn M, Wondimeneh Y, Getaneh A, Ayalew G (2022). Seroprevalence of hepatitis B virus, hepatitis C virus, syphilis and associated factors among female sex workers in Gondar Town, Northwest Ethiopia. Infect Drug Resist.

[31] Tesfa T, Hawulte B, Tolera A, Abate D (2021). Hepatitis B virus infection and associated risk factors among medical students in eastern Ethiopia. PLoS ONE.

[32] Shiferaw Y, Abebe T, Mihret A (2011). Hepatitis B virus infection among medical waste handlers in Addis Ababa, Ethiopia. BioMed Cent Res Notes.

[33] Getie B, Ayalew G, Amsalu A, Ferede G, Yismaw G, Tessema B (2021). Seroprevalence and associated factors of hepatitis B and C virus among pulmonary tuberculosis patients attending health facilities in Gondar Town, Northwest Ethiopia. Infect Drug Resist.

[34] Bane A, Patil A, Khatib M (2014). Healthcare cost and access to care for viral hepatitis in Ethiopia. Int J Innov Appl Stud.

[35] Sheena BS, Hiebert L, Han H, Ippolito H, Abbasi-Kangevari M, Abbasi-Kangevari Z (2022). Global, regional, and national burden of hepatitis B, 1990–2019: a systematic analysis for the global burden of Disease Study 2019. Lancet Gastroenterol Hepatol.

[36] Tordrup D, Hutin Y, Stenberg K, Lauer JA, Hutton DW, Toy M (2019). Additional resource needs for viral hepatitis elimination through universal health coverage: projections in 67 low-income and middle-income countries, 2016–30. The Lancet Global Health.

[37] Easterbrook P, Johnson C, Figueroa C, Baggaley R (2016). HIV and hepatitis testing: global progress, challenges, and future directions. AIDS Rev.

[38] Odimayo MS, Adebimpe WO, Jeff-Agboola YA, Oyeyemi OT, Okiei BN, Adejumo OA (2020). Screening, vaccination, and referrals as viral Hepatitis elimination Triad among internally displaced persons in Edo state, Nigeria. Clin Liver Disease.

[39] Shiferaw F, Letebo M, Bane A (2016). Chronic viral hepatitis: policy, regulation, and strategies for its control and elimination in Ethiopia. BioMed Cent Public Health.

[40] Kang J, Cho JH, Suh CW, Lee DH, Oh HB, Sohn YH (2011). High prevalence of hepatitis B and hepatitis C virus infections in korean patients with hematopoietic malignancies. Ann Hematol.

[41] Omer AR, Salih JI, Al-Nakshabandi AA (2011). Frequency of blood-borne viral infections among leukemic patients in central Iraq. Saudi Med J.

[42] Shaheli M, Yaghobi R, Rezaeian A, Saadi MI, Ramzi M (2015). Study of the associations between TT Virus single and mixed infections with leukemia. Jundishapur J Microbiol.

[43] Torbenson M, Thomas DL (2002). Occult hepatitis B. Lancet Infect Dis.

[44] Raimondo G, Allain JP, Brunetto MR, Buendia M-A, Chen D-S, Colombo M (2008). Statements from the Taormina expert meeting on occult hepatitis B virus infection. J Hepatol.

[45] Raimondo G, Navarra G, Mondello S, Costantino L, Colloredo G, Cucinotta E (2008). Occult hepatitis B virus in liver tissue of individuals without hepatic disease. J Hepatol.

[46] Sagnelli E, Pisaturo M, Martini S, Filippini P, Sagnelli C, Coppola N (2014). Clinical impact of occult hepatitis B virus infection in immunosuppressed patients. World J Hepatol.

[47] Jardim RNCM, Gonçales NSL, Pereira JSF, Fais VC, Gonçales Junior FL (2008). Occult hepatitis B virus infection in immunocompromised patients. Brazilian J Infect Dis.

[48] Zhang Z, Zhang Y, Xu N, Huang C, Li X, Li J (2017). High risk of occult hepatitis B virus infection in leukemia patients from China. Arch Virol.

[49] Schmeltzer P, Sherman KE (2010). Occult hepatitis B: clinical implications and treatment decisions. Dig Dis Sci.

[50] Beykaso G, Mulu A, Giday M, Berhe N, Selamu M, Hailu D et al. Occult Hepatitis B Virus Infection and Its Risks of Cryptic Transmission in Southern Ethiopia. Infection and Drug Resistance. 2022:619 – 30. 10.2147/IDR.S344668 PMC8886027 35241914

[51] Gemechu G, Abagez WE, Alemayehu DH, Tesfaye A, Tadesse D, Kinfu A (2022). Occult hepatitis B virus infection among blood donors in the capital city of Addis Ababa, Ethiopia: implications for blood transfusion safety. Front Gastroenterol.

[52] Power J, El Chaar M, Temple J, Thomas M, Spillane D, Candotti D (2010). HBV reactivation after fludarabine chemotherapy identified on investigation of suspected transfusion-transmitted Hepatitis B virus. J Hepatol.

[53] Marinone C, Mestriner M (2011). HBV disease: HBsAg carrier and occult B infection reactivation in haematological setting. Dig Liver Disease.

[54] Han JW, Yang H, Lee HL, Bae SH, Choi JY, Lee JW (2016). Risk factors and outcomes of hepatitis B virus reactivation in hepatitis B surface antigen negative patients with hematological malignancies. Hepatol Res.

[55] Lau GK, Leung Yh, Fong DY, Au Wy K, Yl, Lie A (2002). High hepatitis B virus (HBV) DNA viral load as the most important risk factor for HBV reactivation in patients positive for HBV surface antigen undergoing autologous hematopoietic cell transplantation. Blood The Journal of the American Society of Hematology.

[56] Weldemhret L, Asmelash T, Belodu R, Gebreegziabiher D (2016). Sero-prevalence of HBV and associated risk factors among HIV positive individuals attending ART clinic at Mekelle hospital, Tigray, Northern Ethiopia. AIDS Res Therapy.

[57] Mohammed H, Eshetie A, Melese D (2022). Prevalence of hepatitis B virus and associated risk factors among adults patients at Dessie referral and Kemise general hospitals in northeastern Ethiopia. Health Sci Rep.

[58] Thanh PN, Tho NTT, Phu TD, Dai Quang T, Duong NT, Chien VC (2020). Prevalence and factors associated with chronic Hepatitis B infection among adults in the Central Highland, Vietnam. AIMS Med Sci.

[59] Montella M, D’Arena G, Crispo A, Capunzo M, Nocerino F, Grimaldi M (2015). Role of sex hormones in the development and progression of hepatitis B virus-associated hepatocellular carcinoma. Int J Endocrinol.

[60] Vom Steeg LG, Klein SL (2016). SeXX matters in infectious disease pathogenesis. PLoS Pathog.

[61] Klein SL, Flanagan KL (2016). Sex differences in immune responses. Nat Rev Immunol.

[62] Fish EN (2008). The X-files in immunity: sex-based differences predispose immune responses. Nat Rev Immunol.

[63] Ben-Batalla I, Vargas-Delgado ME, Von Amsberg G, Janning M, Loges S (2020). Influence of androgens on immunity to self and foreign: effects on immunity and cancer. Front Immunol.

[64] Naugler WE, Sakurai T, Kim S, Maeda S, Kim K, Elsharkawy AM (2007). Gender disparity in liver cancer due to sex differences in MyD88-dependent IL-6 production. Science.

[65] Wang SH, Chen PJ, Yeh SH (2015). Gender disparity in chronic hepatitis B: mechanisms of sex hormones. J Gastroenterol Hepatol.

[66] Benedict N, Samson AA (2015). Seroprevalence of HBV, HCV and HIV infection in patients with haematological malignancies seen at the university of Benin Teaching Hospital, Benin City, Nigeria. IOSR J Dent Med Sci.

[67] Mbanya D, Mikoulou E, Kaptue L (2002). HIV-1 infection in adults with haematological malignancies in Yaounde, Cameroon. West Afr J Med.

[68] Fentaye S, Yibeltal D, Tessema Z. Prevalence of HIV/AIDS Among Elderly People and Associated Factors in Habru Woreda, Amhara Region, Northeast Ethiopia. HIV/AIDS-Research and Palliative Care. 2020;2020:411 – 23. 10.2147/HIV.S265101 PMC7519847 33061656

[69] Tassachew Y, Abebe T, Belyhun Y, Teffera T, Shewaye AB, Desalegn H (2022). Prevalence of HIV and its co-infection with hepatitis B/C virus among chronic liver disease patients in Ethiopia. Hepatic Medicine: Evidence and Research.

[70] Girum T, Wasie A, Lentiro K, Muktar E, Shumbej T, Difer M (2018). Gender disparity in epidemiological trend of HIV/AIDS infection and treatment in Ethiopia. Archives of Public Health.

[71] Ackermann L, Klerk GW (2002). Social factors that make south african women vulnerable to HIV infection. Health Care Women Int.

[72] Kalichman SC, Pellowski J, Turner C (2011). Prevalence of sexually transmitted co-infections in people living with HIV/AIDS: systematic review with implications for using HIV treatments for prevention. Sex Transm Infect.

[73] Frommel D, Teklehaimanot R, Berhe N, Aussel L, Verdier M, Preux PM (1993). A survey of antibodies to hepatitis C virus in Ethiopia. Am J Trop Med Hyg.

[74] Zenebe Y, Mulu W, Yimer M, Abera B (2015). Sero-prevalence and risk factors of hepatitis C virus infection among pregnant women in Bahir Dar city, Northwest Ethiopia: cross sectional study. Pan Afr Med J.

